# Improving Cardiovascular Disease Knowledge among Rural Participants: The Results of a Cluster Randomized Trial

**DOI:** 10.3390/healthcare6030071

**Published:** 2018-06-25

**Authors:** Laurie S. Abbott, Elizabeth H. Slate

**Affiliations:** 1 College of Nursing, Florida State University, Tallahassee, FL 32306-4310, USA; 2 Department of Statistics, Florida State University, Tallahassee, FL 32306-4310, USA; slate@stat.fsu.edu

**Keywords:** community health, health promotion, rural health, cardiovascular disease

## Abstract

Cardiovascular disease (CVD) is a major cause of death and disability, especially among people living in the rural, southern United States. Rural African Americans are often diagnosed with CVD earlier in life, and they bear a disproportionate burden of CVD risk factors, morbidity, and mortality. Health equity among historically underserved, rural populations can potentially be attained through culturally relevant interventions that teach people skills to stay well and avoid CVD-related risk and diagnoses. The purpose of this secondary analysis was to determine the effect of an evidence-based intervention on cardiovascular health knowledge and the stages of change toward the action and maintenance phases. The pre-test-post-test data were obtained during a cluster randomized trial involving twelve rural churches that were randomized to intervention (*n* = 6) and control (*n* = 6) groups. Participants (*n* = 115) in the intervention group received a cardiovascular health intervention, and those (*n* = 114) in the control group could receive the intervention following the study’s completion. The data were analyzed using a linear mixed model to compare group differences from pre-test to post-test. The cardiovascular health promotion intervention significantly improved cardiovascular health knowledge and was associated with advancements in the stages of change toward the action and maintenance phases.

## 1. Introduction

Cardiovascular disease (CVD) is a public health problem that is a major cause of death and disability among people living in the United States [1]. The prevalence of CVD is expected to rise during the next decade because of the aging population and the increased pervasiveness of risk factors, such as too little physical activity, poor diet, uncontrolled hypertension, and diabetes—all of which are modifiable [2]. African Americans bear a disproportionate burden of CVD and an increased risk of hypertension and stroke, and these disparities are especially apparent among those living in the rural southern United States [2,3,4,5,6]. The national objectives listed by Healthy People 2020, a health initiative of the United States government, include improving the overall cardiovascular health among all American people, reducing CVD risk factors, and enhancing the awareness of stroke and heart attack symptoms to stimulate recognition and early medical intervention [7]. In general, African Americans are often diagnosed with CVD at earlier ages, and the early onset of CVD within this population has been attributed to a higher prevalence of CVD risk factors and related adverse health behaviors [8]. Bridging knowledge gaps of CVD through health education interventions is an important step toward achieving these goals and advancing health equity, especially among historically underserved groups [9].

People living in rural areas of the United States have reduced knowledge and awareness about cardiovascular health issues such as knowing the symptoms of a heart attack or a stroke [9,10]. They are also more likely to have low self-efficacy for reading food labels and cooking heart-healthy foods [11]. Limited knowledge and low health literacy about CVD risk factors and associated diagnoses such as heart disease, stroke, and heart failure can hinder CVD risk reduction and the prevention of chronic heart disease exacerbation at home [12,13,14]. Having limited cardiovascular health knowledge is associated with low perceived risk of cardiovascular disease and stroke. For example, African American women living in rural, southern areas of the United States typically understand the causal factors associated with CVD through a combination of learned medical factors and vicarious knowledge that is gained through the experiences of family members and friends that are diagnosed with heart disease [15]. However, having a family history of chronic disease, such as stroke, does not predict improved health knowledge and habits, such as a good diet and exercise [12].

Educational interventions that are designed to improve knowledge, heart health habits, and related skills are crucial for advancing cardiovascular health equity among people living in underserved areas in the rural, southern United States [5,12,13,14]. Research is needed to determine whether educational interventions increase the knowledge that is necessary to promote health behavior changes and to reduce the overall cardiovascular disease risk [12]. The purpose of this secondary analysis was to examine the effects of the culturally relevant With Every Heartbeat is Life intervention on cardiovascular health knowledge [16]. The Integrated Model of Behavioral Prediction was the theoretical framework that was used to guide the study [17]. This health behavior model explains the relationships among factors such as norms, attitudes, and self-efficacy in regard to intentions to adopt recommended health practices [17]. Health knowledge fosters essential health skills that are associated with progressing intentions toward active health behavior performance [17]. 

## 2. Materials and Methods

The current study is a secondary analysis that examined data that was collected during a cluster randomized trial among African American participants living in the rural, southeastern United States. The parent study had a pre-test-post-test strategy that observed the intervention effects of a health promotion program among the participants who were recruited from randomized churches that were located in two rural counties. Detailed information about the methods, recruitment strategies, and sample size calculations that were used during the parent study has been previously published in a manuscript that described the effect of the intervention on psycho-social aspects such as intentions, norms, attitudes, and self-efficacy [18]. The procedures and human ethics of this secondary data analysis that analyzed knowledge and stage of change variables were reviewed and approved by the institutional review board (IRB) of Florida State University.

### 2.1. Sample and Setting

The participants (*n* = 229) in the parent study were recruited from twelve rural churches that were randomized to intervention (*n* = 6) and control (*n* = 6) groups. Church settings have been found to be a cultural strength within an already established community that facilitates reaching African American groups and may influence participation in health behavior change interventions [8,11]. Of the total 229 participants, 115 people were in the intervention group and 114 participants were in the control group. Eligible participants were (a) men and women who self-identified as African American, (b) at least 24 years of age, and (c) able to read, write, and understand English. The informed consent forms were signed after all questions about the study were satisfactorily answered. The participants from churches that were randomized to the intervention group received the heart health curriculum from the same public health nurse. The pastors of the control group churches were given the option of having the intervention delivered in their churches following the completion of the study. A US $20 gift card incentive was issued to the participants in both the intervention and the control groups during the final data collection period. 

### 2.2. Intervention 

The participants in the churches who were randomized to the intervention group received the With Every Heartbeat is Life cardiovascular health promotion program [16]. The curriculum was developed and culturally tailored specifically for African American groups by the National Heart, Lung, and Blood Institute (NHLBI). Although it was designed to be implemented over ten, weekly sessions of approximately 45 min to an hour each, we adapted the program for delivery in six, weekly sessions, each lasting about ninety minutes to accommodate schedule logistics. Related topics were combined into one session, and the time frame for each session was lengthened to include the increased educational material and interactive activities. This adaptation was done in response to feedback from the church group representatives that the initially planned twelve-session intervention, including two additional days for pre-test and post-test data collection, was too lengthy and would result in reduced participation. The weekly topics addressed major CVD risk factors such as diabetes, hypertension, diet, elevated serum cholesterol, excessive weight, physical inactivity, and smoking. 

### 2.3. Measures

The data used in the secondary analysis were collected during two time periods. Within the intervention group, data were collected at baseline and after the sixth week’s session. Within the control group, the data were collected at baseline and six weeks later. The program-specific “My Health Knowledge” instrument had twenty-one items for measuring cardiovascular health knowledge [19]. There was one item that measured participant movement toward an action phase or stage of change. 

Heart health knowledge. The “My Health Knowledge” instrument measured participant knowledge about cardiovascular health topics: heart disease risk, heart attack and stroke symptoms, diet including cholesterol, diabetes, weight management, smoking, and the effects of alcohol. Similar items within the measure were evaluated by adapting a method from a previous study using an earlier, yet similar version of the instrument that was published for use with the With Every Heartbeat is Life curriculum [20]. The categories are (a) Risk Factors, (b) Disease Symptoms, (c) Risk Reduction, and (d) Heart Health Facts. For example, the Risk Factors section includes questions about factors that influence cardiovascular disease risk. The Disease Symptoms area has items for measuring knowledge about the signs and symptoms of diabetes, heart attack, and stroke. The Risk Reduction category asks about strategies for reducing cardiovascular disease risk, and the Heart Health facts includes basic information about cardiovascular disease issues, such as the parameters for normal blood pressure, blood glucose, and blood cholesterol levels. The combined value of all of the items in the “My Health Knowledge” measure equaled 100 points, and the summed participant responses were indicative of the percentage of correctly answered items.

Stages of change. There was one item on the “My Health Knowledge” instrument that was titled, “A Day with the Harris Family” that measured the participants’ readiness to make cardiovascular health habit changes. The item was based on the 5-stage continuum that was described by the Trans-Theoretical Model: precontemplation, contemplation, preparation, action, and maintenance [21]. A scenario was provided that described the situation, and the answer options included five fictitious people who represented one of the five stages of change. For example, choosing Ms. Diane, “I am taking action.”, signified the action stage. Using the classification method which was adapted from a previous publication by Hurtado et al., the 5-point measure was dichotomized by combining the first three stages (precontemplation, contemplation, and preparation) and the last two stages (action and maintenance) [20].

### 2.4. Data Analysis

The socio-demographic characteristics of the participants were described using frequencies, averages, and standard deviations. The statistical procedure that was used to assess group differences from pre-test to post-test was the significance of the interaction between time and group assignment in a repeated measures linear mixed model (LMM) using the mixed procedure. The model included fixed effects for study group assignment, time, and the time-by-group interaction, together with a random effect for church—the last of which accommodated the within-cluster correlation among the responses. All of the analyses used the intention-to-treat paradigm in which all participants were included in the group to which their church was randomized. Missing post-test responses (*n* = 12 and *n* = 4 in the intervention and control groups, respectively) were handled via a maximum likelihood estimation of the LMM. Results are presented as point estimates and confidence intervals for the time-by-group interaction effect and the changes from pre-test to post-test for the two groups. No multiplicity adjustment was used. Analyses were performed using the mixed procedure in IBM SPSS Statistics, version 22 (IBM, Armonk, NY, USA).

## 3. Results

There were no substantial group differences regarding socio-demographic characteristics such as gender, age, educational attainment, and employment levels (Table 1). The results of the secondary analysis indicated that participation in the intervention was associated with cardiovascular health knowledge improvements. Compared with the control group, the intervention group had statistically significant overall differences (*p* < 0.001) from pre-test to post-test (Table 2). The results for the overall test were summed from the individual items, with 100 being the best possible score. The mean (*M*) baseline or pre-test scores for both the intervention (*M* = 78.03) and control (*M* = 78.86) groups were similar. However, the mean post-test score for the intervention group (*M* = 94.52) was substantially higher than the mean post-test score (*M* = 80.86) for the control group. The findings for each classification are listed with detailed descriptions about the items that were included within each of the categorical headings. 

### 3.1. Knowledge of Risk Factors

The results of the overall score for the Risk Factors knowledge category showed statistically significant (*p* < 0.001) group differences (Table 2). Within this category, five of the six individual items about risk factor topics that had statistically significant results were (a) general heart disease risk (*p* = 0.005), (b) cholesterol levels (*p* = 0.044), (c) risk for diabetes (*p* = 0.004), (d) smoking as a chronic disease risk factor (*p* < 0.001), and e) blood pressure increased by alcohol consumption (*p* < 0.001). There was one item within this topic grouping about second-hand smoke as a risk factor for heart and lung disease that showed no significant knowledge improvement (*p* = 0.117).

### 3.2. Disease Symptoms

There were statistically significant group differences (*p* < 0.001) for the overall score in the Disease Symptoms category. All of the individual items within this classification had statistically significant results including (a) diabetes symptoms (*p* = 0.006), (b) stroke signs (*p* < 0.001), and (c) heart attack signs (*p* < 0.001). 

### 3.3. Risk Reduction 

There were statistically significant findings (*p* = 0.009) for the overall score in the Risk Reduction category. All of the individual items that were grouped in this category had significant results, and these were (a) heart disease risk reduction (*p* = 0.012), (b) weight loss (*p* = 0.034), (c) exercise (*p* = 0.034), and (d) minimal time each day that should be spent exercising (*p* = 0.034).

### 3.4. Heart Health Facts

There were statistically significant results (*p* < 0.001) for the overall score in the Heart Health Facts category. The statistically significant individual items within this grouping were general facts about heart health, such as (a) heart attack facts (*p* < 0.001), (b) vegetable servings per day (*p* < 0.001), (c) women’s waist measurement (*p* = 0.001), (d) men’s waist measurement (*p* < 0.001), (e) blood pressure reading (*p* = 0.003), (f) cholesterol levels (*p* < 0.001), and (g) blood glucose levels (*p* < 0.001). The one item that was not statistically significant between groups was about blood pressure being a silent killer (*p* = 0.182).

### 3.5. Stage of Change

The one item within the Stage of Change category indicated that there were statistically significant changes from baseline (*p* < 0.001) between the groups. For Stage of Change, the proportion of participants either taking action or maintaining a healthy path was similar at pre-test for the control and the intervention groups at 53.5% and 50.4%, respectively. At post-test, these proportions increased to 59.1% and 80.6%, respectively.

## 4. Discussion

The results of this secondary analysis indicate that the educational intervention in a rural community setting was useful for improving cardiovascular knowledge and may promote healthier lifestyle choices and behaviors. Compared with the control group, the participants in the intervention group had significantly improved knowledge associated with CVD, risk reduction strategies, and recognizing the signs and symptoms of cardiovascular events such as heart attack and stroke. For the people in the intervention group, participation in the intervention significantly influenced stage of change toward the action and maintenance phases. Theoretically, movement toward the action phase indicates active performance of a recommended health behavior, and the maintenance phase involves sustained behavior over time [21]. Having increased knowledge about CVD pathophysiology, related CVD risk factors, and prevention strategies using understandable language, culturally relevant examples, and skill-building activities such as role-plays and label-reading exercises may have influenced the intervention group participants and motivated them to actively engage in the recommended cardiovascular health behaviors.

There were two items that did not demonstrate statistically significant changes in participant knowledge from baseline to post-test. The items that measured knowledge about the dangers of second-hand smoke exposure (*p* = 0.117) and blood pressure (*p* = 0.182) had no significant between-group post-test differences. An influencing factor may have been that the two questions asked about topics that are considered simple knowledge, meaning the participants had high scores at baseline, leaving little room for improvement. The smoking question asked whether second-hand smoke was associated with increased heart and lung disease, and the question about blood pressure asked whether hypertension was considered a “silent killer” because people do not recognize the symptoms. Further, both questions had “Yes”, “No”, or “Don’t Know” answer options which made guessing the correct answer easier. 

The findings of other studies indicate that evidence-based guidelines that incorporate cultural preferences and attitudes can positively influence health behavior modifications among African American populations [8]. Educational health programs have typically been effective strategies for improving knowledge about CVD, which is necessary for making heart healthy choices and reducing modifiable CVD risk factors [22]. Such interventions are crucial for bridging knowledge gaps among people at increased risk for adverse cardiovascular outcomes [9]. For example, the outcomes of a nutritional intervention study included improvements on weight and blood pressure parameters, increased produce consumption, and reduced intake of overly processed foods with a high sodium content [23]. A different intervention showed that an educational intervention and access to produce in a community garden had greater improvements in produce consumption than access to the community garden alone [24]. Educational interventions can empower participants to better care for themselves and make healthier lifestyle choices that are associated with decreased risk reduction [11]. The outcomes of an educational intervention among minority women increased their knowledge about heart disease and stroke, CVD risk factors, and taking appropriate action for symptom presentation [9]. A stroke prevention program implemented among African American participants in rural churches had the positive effects of increasing CVD knowledge and reducing blood pressure measures [25]. Another intervention in rural African American church settings successfully improved awareness about CVD and the need for eating a heart-healthy diet and increasing physical activity levels [26].

A limitation of the study is the narrow geographical location from which the participants were recruited, meaning that the results may not be generalizable to other areas. The implementation of the study in two neighboring rural counties could have increased the possibility of cross-contamination between the two study groups. Additionally, the aim of the study design was to analyze the intervention effect on knowledge from pre-test to post-test, however it did not evaluate whether knowledge predicted health behavior changes. Another limitation was that the study did not address whether the knowledge was retained over longer lengths of time. Future research efforts could determine the long-term effects of increased cardiovascular knowledge on lifestyle choices and improvements in biological parameters such as weight, blood pressure, and plasma cholesterol levels.

## 5. Conclusions

A culturally relevant health education intervention designed to improve CVD knowledge can potentially advance health equity and improve cardiovascular health outcomes among underserved populations. African Americans living in the rural, southern United States are disproportionately burdened by CVD and related chronic diseases and have been historically difficult to engage in health promotion research efforts. The positive results of this study support future efforts targeting CVD risk reduction and health knowledge improvement efforts within rural communities. Public health nurses are particularly well-suited to implement evidence-based health promotion programs in remote community settings and to participate in health disparity research efforts.

## Figures and Tables

**Table 1:** Sociodemographic characteristics of the sample.

Demographic Variable		Intervention Group (*n* = 115)				Control Group (*n* = 114)			
		*n*	%	*M*	*SD*	*n*	%	*M*	*SD*
Age (years)				59.03	12.91			56.56	13.49
Race									
	African American	115	100			114	100		
Gender									
	Male	31	27.0			35	30.7		
	Female	84	73.0			79	69.3		
Educational level									
	Did not finish high school	21	18.3			22	19.3		
	Graduated from high school/General Education Diploma (GED)	28	24.3			45	39.5		
	Attended some college	32	27.8			23	20.0		
	Graduated from college	23	20.0			14	12.3		
	Earned a graduate/professional degree	11	9.6			10	8.8		
Employment Status									
	Employed (Full-time or Part-time)	53	46.1			59	51.8		
	Not Employed (Retired/Homemaker or Unemployed)	62	54			55	48.2		

Note: The entries provided are counts (*n*) and percentages (%) except for age (*M* = average, *SD* = standard deviation).

**Table 2:** A comparison of study outcomes for the intervention and control groups.

Variable	Control Group *		Intervention Group ^+^		Intervention Effect ^±^		
	Δ_C_	95% CI	Δ_I_	95% CI	*b*	95% CI	*p*
My Health Knowledge (MHK), Overall	2.00	(−1.57, 5.58)	15.50	(12.84, 20.16)	14.49	(9.38, 19.61)	0.000
Risk Factors (RF)							
RF, Overall	1.043	(−0.343, 2.429)	5.395	(3.978, 6.811)	4.352	(2.370, 6.333)	0.000
HDRisk1	0.412	(−0.157, 0.982)	1.580	(0.998, 2.160)	1.17	(0.353, 1.98)	0.005
Cholest2	0.300	(−0.034, 0.634)	0.790	(0.449, 1.131)	0.490	(0.013, 0.967)	0.044
DiaRisk3	0.423	(−0.013, 0.859)	1.335	(0.891, 1.780)	0.913	(0.289, 1.536)	0.004
SmRisk9	−0.159	(−0.643, 0.324)	1.318	(0.825, 1.811)	1.477	(0.787, 2.168)	0.000
Alcoh19	0.063	(−0.031, 0.156)	0.343	(0.248, 0.439)	0.280	(0.147, 0.414)	0.000
Smok20	0.005	(−0.043, 0.054)	0.061	(0.011, 0.110)	0.055	(0.014, 0.125)	0.117
Disease Symptoms (DS)							
DS, Overall	0.709	(−0.527, 1.946)	4.605	(3.342, 5.868)	3.900	(2.129, 5.663)	0.000
DiaSym4	0.238	(−0.167, 0.642)	1.053	(0.640, 1.466)	0.815	(0.237, 1.393)	0.006
StrSigns5	0.145	(−0.444, 0.734)	1.862	(1.260, 2.463)	1.716	(0.874, 2.558)	0.000
HASigns6	0.324	(−0.186, 0.834)	1.700	(1.179, 2.220)	1.375	(0.647, 2.104)	0.000
Risk Reduction (RR)							
RR, Overall	0.235	(−0.963, 1.433)	2.535	(1.313, 3.757)	2.300	(0.588, 4.011)	0.009
HDRiskRed8	0.185	(−0.308, 0.678)	1.095	(0.591, 1.600)	0.910	(0.205, 1.615)	0.012
WeiLoss10	0.026	(−0.428, 0.481)	0.728	(0.266, 1.190)	0.702	(0.053, 1.350)	0.034
Exerc11	0.026	(−0.428, 0.481)	0.728	(0.266, 1.190)	0.702	(0.053, 1.350)	0.034
MinEx15	−0.019	(−0.111, 0.073)	0.123	(0.029, 0.216)	0.142	(0.011, 0.273)	0.034
Heart Health Facts (HHF)							
HHF, Overall	−0.042	(−0.472, 0.388)	2.645	(2.207, 3.084)	2.687	(2.073, 3.301)	0.000
HAFacts7	0.061	(−0.328, 0.450)	1.416	(1.020, 1.813)	1.356	(0.800, 1.911)	0.000
VeServ12	0.000	(−0.106,0.106)	0.290	(0.182, 0.398)	0.290	(0.139, 0.441)	0.000
WomWai13	0.081	(−0.019, 0.180)	0.325	(0.224, 0.427)	0.245	(0.102, 0.387)	0.001
MenWai14	0.010	(−0.092, 0.113)	0.485	(0.380, 0.589)	0.474	(0.328, 0.621)	0.000
BP16	−0.076	(−0.185, 0.032)	0.163	(0.052, 0.274)	0.240	(0.275, 0.936)	0.003
Chol17	−0.030	(−0.261, 0.202)	0.576	(0.341, 0.812)	0.606	(−0.055, 0.338)	0.000
BlGlu18	−0.047	(−0.218, 0.124)	0.726	(0.552, 0.900)	0.773	(0.529, 1.017)	0.000
BP21	0.016	(−0.040, 0.071)	0.070	(0.013, 0.126)	0.054	(−0.025, 0.133)	0.182
Stage of Change (SC)							
SC	0.022	(−0.246, 0.203)	0.651	(0.422, 0.881)	0.673	(0.352, 0.994)	0.000

* Δ_C_ is the pre-test to post-test change for the control group, as estimated from the LMM. ^+^ Δ_I_ is the pre-test to post-test change for the intervention group, as estimated from the LMM. ^±^ *b* is the estimate of the effect of the intervention, i.e., the estimate of the coefficient for the interaction between time (pre-test to post-test) and study group in the LMM (also, *b* = Δ_I_ − Δ_C_).
